# Supplementary material for: Reliable Flow-Cytometric Approach for Minimal Residual Disease Monitoring in Patients with B-Cell Precursor Acute Lymphoblastic Leukemia after CD19-Targeted Therapy
Source: Cancers (Basel). 2022 Nov 5;14(21):5445. doi: 10.3390/cancers14215445 (PMC9658935; doi:10.3390/cancers14215445)
Supplement: Supplementary file 1 [file cancers-14-05445-s001.zip › cancers-1988076-supplementary.pdf]

Table S1. Characteristics of patients tested for MRD during the approbation phase

**n**<br>**Sex, m/f**	65<br>38/27
**Age**	9.5 years (range 0.9 - 20.7 years)
**Diagnosis**<br>BI-ALL<br>BII-ALL<br>BIII-ALL<br>BIV-ALL	 8<br>55<br>1<br>1
**Chromosomal aberration**<br>*KMT2A* rearranged<br>*TCF3* rearranged<br>t(12;21)(p13;q22)/*ETV6-RUNX1*<br>*IgH* rearranged<br>*CRLF2* rearranged<br>Complex karyotype<br>t(9;22)(q34;q11)/*BCR-ABL1*<br><br>Hyperdiploid<br>Hypodiploid<br><br>Other aberrations (Intrachromosomal amplification of *RUNX1*, del9p, *PDGFRbeta* rear, *ABL1* rear, trisomy 21)<br><br>No well-established chromosomal aberrations<br>No data	58/64 (90,6%)<br>10<br>8<br>7<br>5<br>5<br>2<br>2<br><br>11<br>2<br><br>6<br><br>6<br>1

Table S2. FISH probes used for initial molecular genetic diagnostics

**Entity**	**FISH probes**
Hyperdiploidy, hypodiploidy	MetaSystems XCE 4/10/17
KMT2A rearrangement	Vysis LSI KMT2A break-apart
t(1;19)(q23;p13)/*TCF3::PBX1*	Vysis LSI TCF3/PBX1 DCDF
t(17;19)(q22;p13)/*TCF3::HLF*	Cytocell E2A break-apart<br>CytoCell E2A (TCF3)/PBX1 Plus
t(12;21)(p13;q22)/*ETV6::RUNX1*, amp*RUNX1*	Vysis LSI ETV6/RUNX1 DF
t(9;22)(q34;q11)/*BCR::ABL1*	Vysis LSI BCR/ABL DCDF
CRLF2 rearrangement	Kreatech ON CRLF2(Xp22/Yp11)/ IGH<br>Cytocell P2RY8 break-apart<br>Cytocell CRFL2 break-apart<br>Vysis LSI IGH break-apart

Vysis probes are of Abbott Molecular, Des Plaines, Illinois, USA
MetaSystems probes are of MetaSystems GmbH, Altlussheim, Germany
Kreatech probes are of Leica Microsystems B.V., Amsterdam, Netherlands
Cytocell probes are of Cytocell, Milton, Cambridge, UK

Table S3. Custom primer pairs used for RT-PCR

**Entity**	**GenBank accession number**	**Primer pair**
t(1;11)(p32;q23)/*KMT2A::EPS15*	DNA MN788376	KMT2A-F 5’-AGGAGAATGCAGGCACTTTGA-3’ EPS15-R 5’-AGCATCAGAAGCCAACACCC-3’
t(17;19)(q22;p13)/*TCF3::HLF*	DNA MW400960 RNA MW400957	E2A-F/43758 5’-TCGCCCAGCTACGACGGGGGTCTC-3’ HLF-R/43758 5’-GAGGCCCGGATGGCGATCTG-3’
t(17;19)(q22;p13)/*TCF3::HLF*	DNA MW400959 RNA MW400956	E2A-F/33863 5’-TCGCCCAGCTACGACGGGGGTCTC-3’ HLF-R/33863 5’-GAGGCCCGGATGGCGATCTG-3’

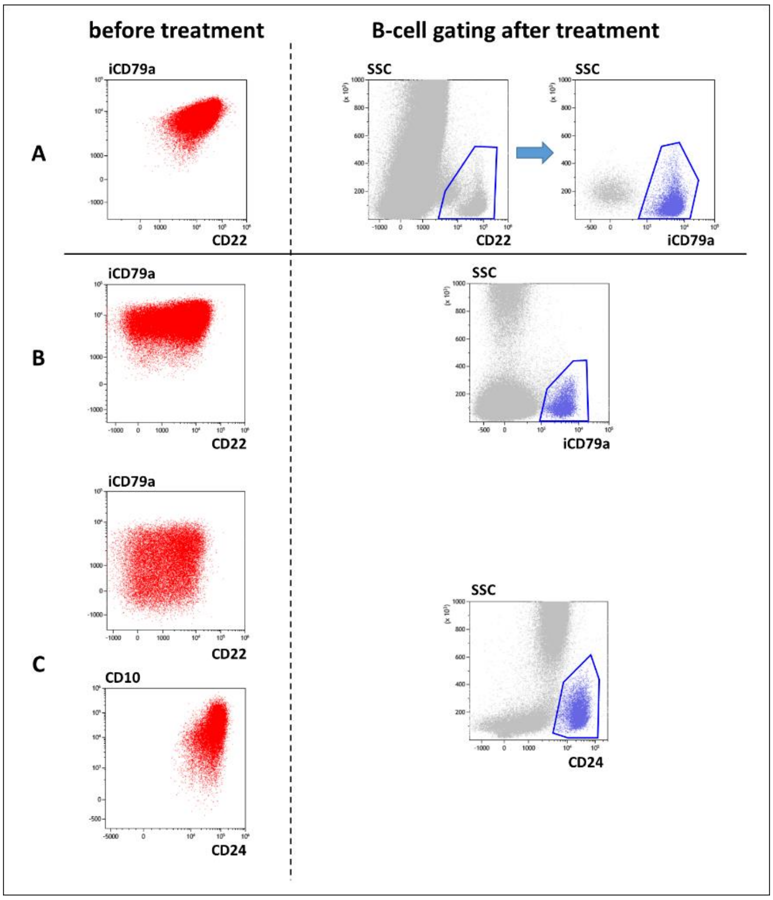

Figure S1. Examples of B-lineage compartment gating in follow-up bone marrow samples based on the initial antigens expression. **Panel A** shows the most widespread gating sequence (total initial positivity for CD22 and iCD79a). **Panel B** shows secondary gating algorithm (heterogeneous CD22 expression with total positivity for intracellular (i) CD79a). **Panel C** depicts the rarest gating sequence applied because of heterogeneous expression of both CD22 and iCD79a. Cells in the final B-lineage gate are blue, leukemic blasts are red, remaining cells are grey.

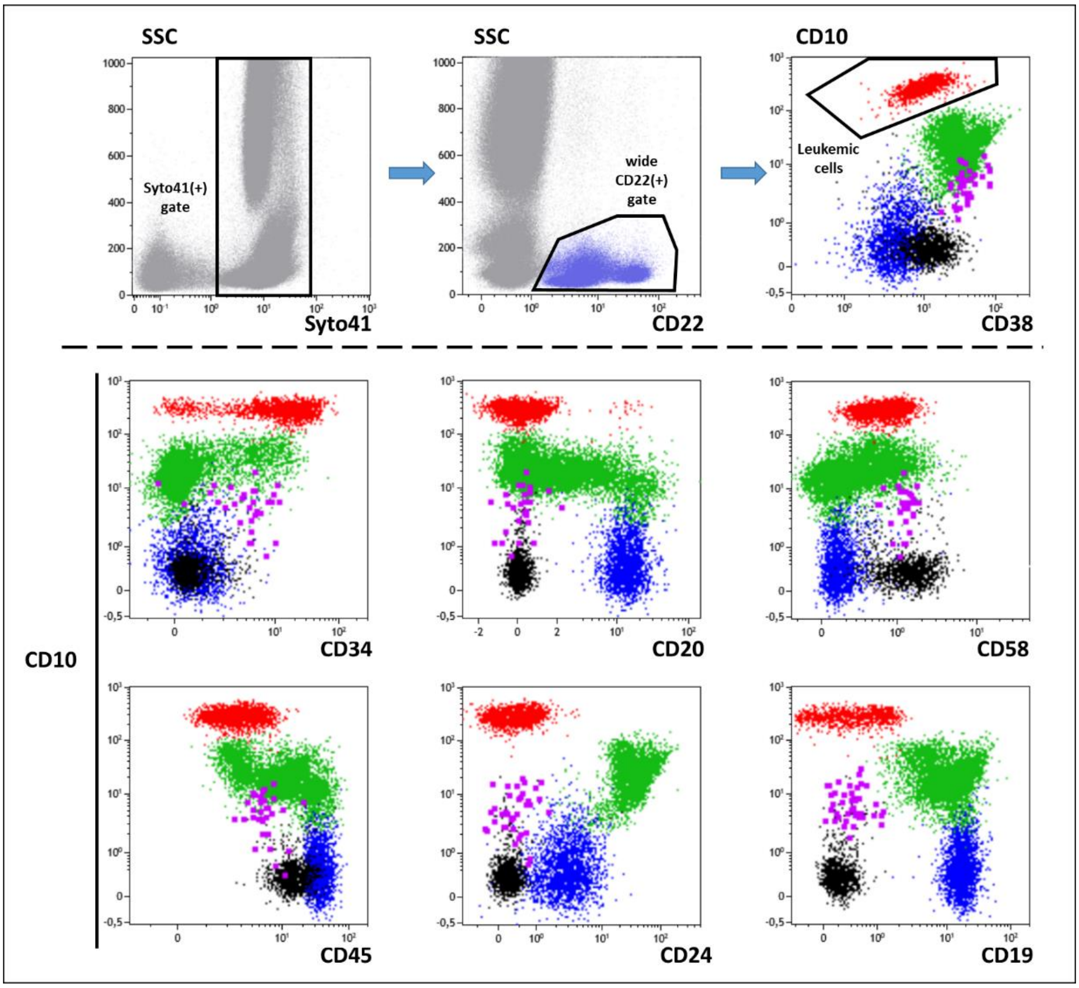

Figure S2. Example of MRD data analysis using CD22 as marker for B-cell gating. First row contains sequence of the search for leukemic cells on dot plots, second and third rows demonstrate localization of CD22(+) cells on dot plots. Blue, cells in B-cell gate; red, leukemic cells; green, CD19(+) BCPs; purple, CD19(−) BCPs; black, basophils or plasmacytoid dendritic cells; dark blue, mature B-cells; grey, other cells.

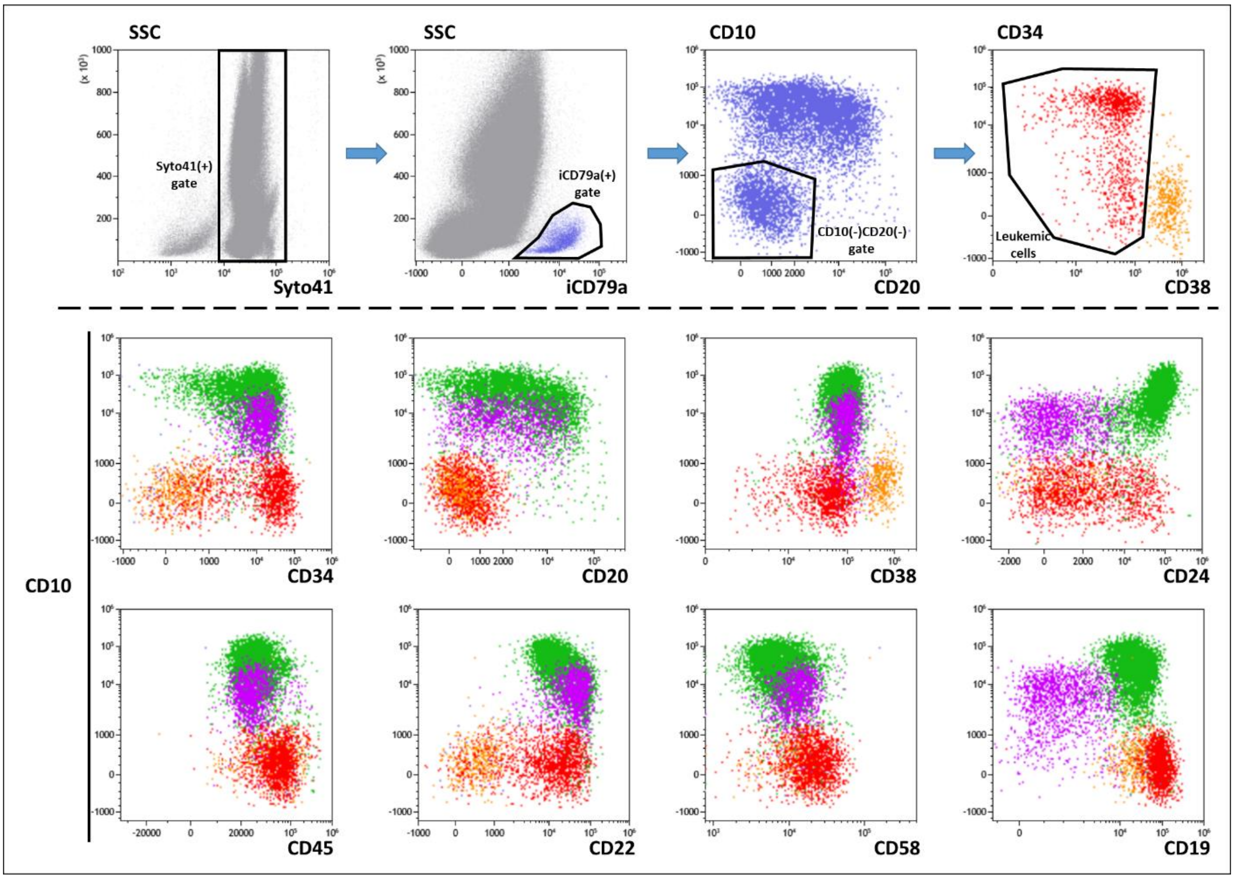

Figure S3. Example of MRD data analysis using iCD79a as marker for B-cell gating. First row contains sequence of the search for leukemic cells on dot plots, second and third rows demonstrate localization of iCD79a(+) cells on dot plots. Blue, cells in B-cell gate; red, leukemic cells; green, CD19(+) BCPs; purple, CD19(−) BCPs; orange, plasma cells; grey, other cells.

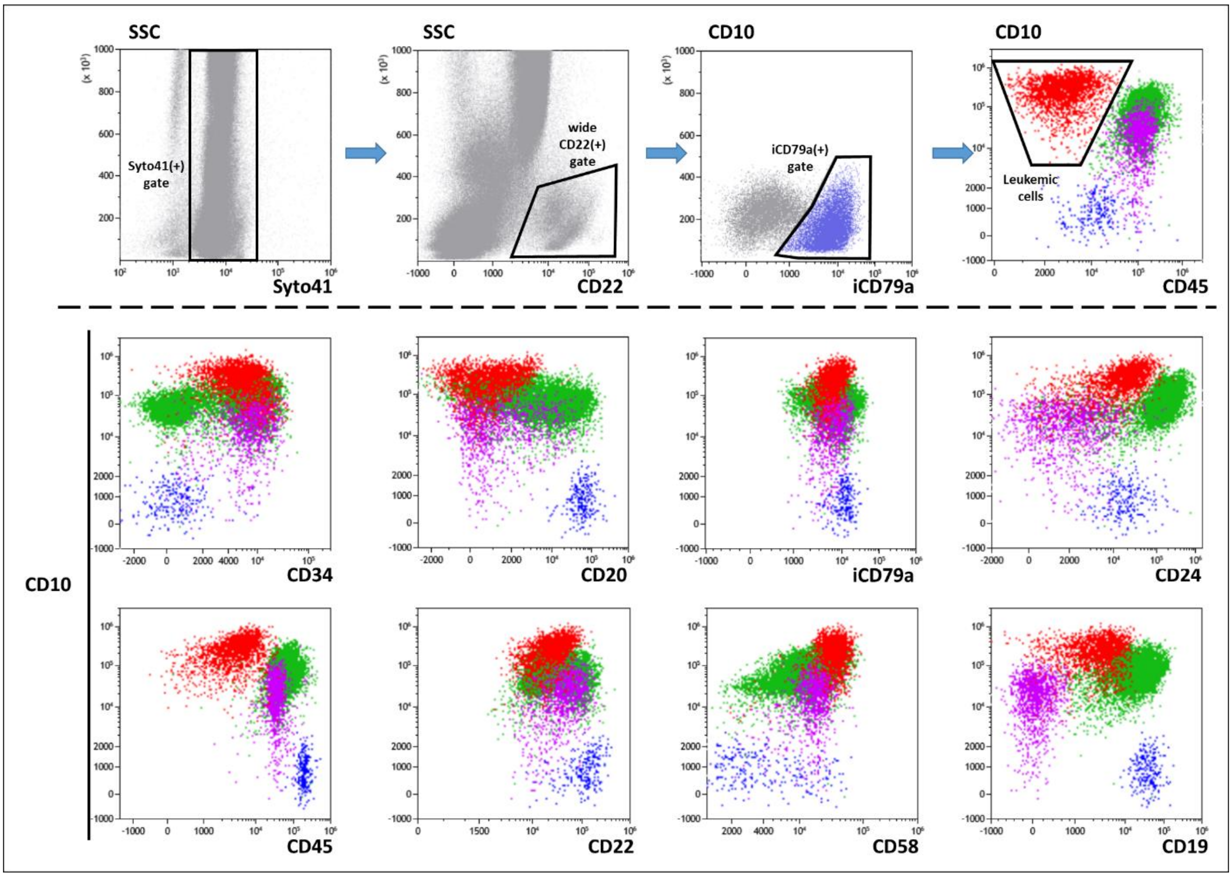

Figure S4. Example of MRD data analysis using combination of CD22 and iCD79a for B-cell gating. First row contains sequence of the search for leukemic cells on dot plots, second and third rows demonstrate localization of CD22(+)iCD79a(+) cells on dot plots. Blue, cells in B-cell gate; red, leukemic cells; green, CD19(+) BCPs; purple, CD19(−) BCPs; dark blue, mature B-cells; grey, other cells.

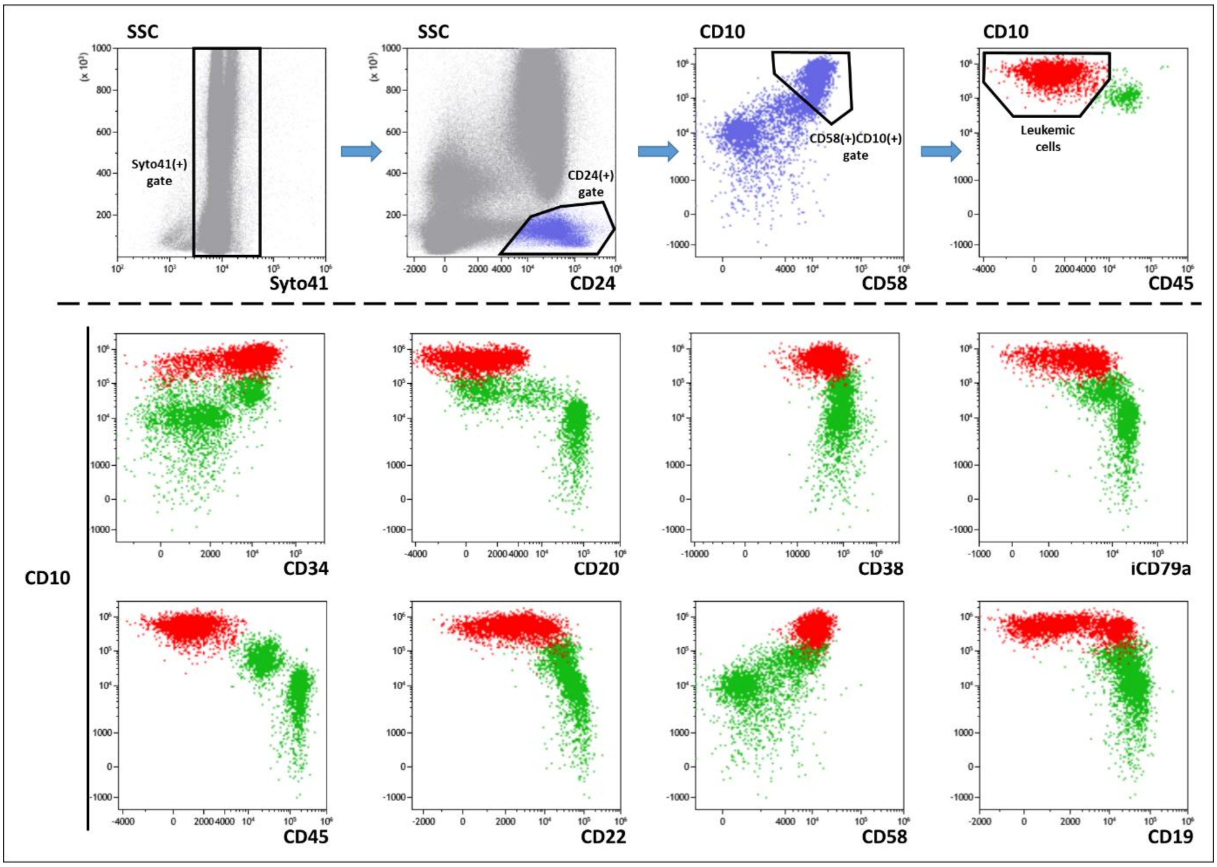

Figure S5. Example of MRD data analysis using CD24 as marker for B-cell gating. First row contains sequence of the search for leukemic cells on dot plots, second and third rows demonstrate localization of CD24(+) cells on dot plots. Blue, cells in B-cell gate; red, leukemic cells; green, CD19(+) BCPs; grey, other cells.
